# Development and External Validation of an Improved Version of the Diagnostic Model for Opportunistic Screening of Malignant Esophageal Lesions

**DOI:** 10.3390/cancers14235945

**Published:** 2022-11-30

**Authors:** Zhen Liu, Hongchen Zheng, Mengfei Liu, Yujie He, Yun Chen, Ping Ji, Zhengyu Fang, Ping Xiao, Fenglei Li, Chuanhai Guo, Weihua Yin, Yaqi Pan, Zhonghu He, Yang Ke

**Affiliations:** 1 Key Laboratory of Carcinogenesis and Translational Research (Ministry of Education/Beijing), Laboratory of Genetics, Peking University Cancer Hospital & Institute, Beijing 100142, China; 2 Endoscopy Center, Hua County People’s Hospital, Hua County 456483, China; 3 Department of Ultrasound, Peking University Shenzhen Hospital, Shenzhen 518034, China; 4 Shenzhen Key Laboratory for Drug Addiction and Medication Safety, Shenzhen Peking University-Hong Kong University of Science and Technology Medical Center, Shenzhen 518034, China; 5 Clinical Research Institute, Shenzhen Peking University-Hong Kong University of Science and Technology Medical Center, Shenzhen 518034, China; 6 Hua County People’s Hospital, Hua County 456483, China; 7 Department of Pathology, Peking University Shenzhen Hospital, Shenzhen 518034, China

**Keywords:** esophageal squamous cell carcinoma, opportunistic screening, diagnostic model, model updating, external validation

## Abstract

**Simple Summary:**

Simple and effective risk stratification tools which allow prediction of the risk of malignant esophageal lesions are needed for the practice of opportunistic screening. The aim of the current study was to develop an improved version of the diagnostic model based on a large-scale outpatient cohort and assess the robustness and generalizability of the model through external validations. The improved diagnostic model had seven predictors and generated an area under the receiver operating characteristic curve of 0.860 in the development set. Validation of the model in two external populations also showed high discrimination power and was able to increase the detection rate of malignant esophageal lesions. This questionnaire-based diagnostic model provides an easy-to-use tool to identify high-risk individuals and will be useful for the promotion of the opportunistic screening of esophageal cancer.

**Abstract:**

We aimed to develop an improved version of the diagnostic model predicting the risk of malignant esophageal lesions in opportunistic screening and validate it in external populations. The development set involved 10,595 outpatients receiving endoscopy from a hospital in Hua County, a high-risk region for esophageal squamous cell carcinoma in northern China. Validation set A enrolled 9453 outpatients receiving endoscopy in a non-high-risk region in southern China. Validation set B involved 17,511 residents in Hua County. The improved diagnostic model consisted of seven predictors including age, gender, family history of esophageal squamous cell carcinoma, smoking, body mass index, dysphagia, and retrosternal pain, with an area under the receiver operating characteristic curve (AUC) of 0.860 (95% confidence interval: 0.835–0.886) in the development set. Ideal discrimination ability was achieved in external validations (AUC _validation set A_: 0.892, 95% confidence interval: 0.858–0.926; AUC _validation set B_: 0.799, 95% confidence interval: 0.705–0.894). This improved model also markedly increased the detection rate of malignant esophageal lesions compared with universal screening, demonstrating great potential for use in opportunistic screening of malignant esophageal lesions in heterogeneous populations.

## 1. Introduction

Esophageal cancer (EC) is one of the most common cancers and is the leading cause of cancer death globally [1,2]. There were an estimated ~600,000 new cases and ~540,000 deaths worldwide in 2020 [1]. More than half of the world’s new EC cases occurred in China [3]. Esophageal squamous cell carcinoma (ESCC) was the predominant histologic type [3].

Due to the lack of typical symptoms at an early stage, most ESCC cases are diagnosed at an advanced stage with low 5-year survival [4]. Great importance has therefore been attached to screening. Cancer screening programs can be classified into organized screening and opportunistic screening according to differences in decision-maker, implementation, and payers. Organized screening programs for EC have been implemented by the government in high-risk areas in China. However, this screening modality entails continuous massive investment in human and material resources and is difficult to expand and sustain on a large scale. In contrast, opportunistic screening, which is defined as screening for patients who present to healthcare professionals for any complaint, is more cost-effective and preferable for scaling up esophageal cancer screening [5].

For the implementation of opportunistic screening for ESCC, two prerequisites must be considered. First, there must be confirmed evidence supporting the effectiveness of screening. Observational studies have reported that early-stage EC patients can benefit from cancer screening [6,7,8], and large-scale randomized controlled trials (RCT) have been initiated to provide the highest-grade evidence in the future [9,10]. Second, a simple and effective risk stratification tool to predict the risk of malignant esophageal lesions is needed to help patients and physicians decide whether to accept endoscopic examinations and to guide endoscopists.

In a previous study, we constructed the first model predicting the risk of prevalent esophageal malignant lesions for ESCC opportunistic screening by combining five easy-to-collect predictors [11]. That model showed good discrimination in the development set. More validations are needed to evaluate the performance of that model in real-world screening scenarios and heterogeneous populations. Therefore, in this study, we aimed to develop an improved version of the model using a larger development set, and to assess the robustness and generalizability of the model through external validation using two different cohorts. We further tested its ability of increasing the detection rate of opportunistic screening.

## 2. Materials and Methods

### 2.1. Study Participants

#### 2.1.1. Development Set

The development set used for the original model consisted of outpatients receiving upper gastrointestinal (GI) endoscopy at the Hua County People’s Hospital from 1 March 2017 to 20 February 2019. This hospital is located in Henan Province, which is a high-risk region in northern China [11]. In the current study, we expanded the development set by additionally enrolling consecutive outpatients from 21 February 2019 to 31 December 2021 and constructed an improved version of the model.

#### 2.1.2. Validation Set A

Validation set A was previously used for validation of the original model and enrolled outpatients undergoing endoscopy in Peking University Shenzhen Hospital from 19 June 2017 to 14 January 2019. This hospital is a tertiary hospital in Shenzhen, which is an economically dynamic city with a huge migrant population and a low incidence of ESCC in southern China [11]. In this study, validation set A was expanded by recruiting additional consecutive outpatients up to 18 November 2021.

#### 2.1.3. Validation Set B

To assess the performance of the model in a general population, we further validated it in the control group of the Efficacy of endoscopic Screening for Esophageal Cancer in China (ESECC) trial (ClinicalTrial: No. NCT01688908) as validation set B. As described previously, ESECC is a randomized controlled trial conducted in Hua County to evaluate the efficacy and cost-effectiveness of endoscopic screening for EC [9]. A total of 668 villages were randomly selected and equally allocated to a screening group and a control group [9]. The participants in the control group did not receive endoscopic screening.

#### 2.1.4. Inclusion Criteria

For all three datasets, inclusion criteria were: (1) age 45 to 69 years; (2) no history of cancer, mental disorder, or contraindications for endoscopy; and (3) completion of an adequate upper GI endoscopic examination (not applicable for validation set B).

### 2.2. Data Collection and Outcome Ascertainment

All participants in these three cohorts completed a one-on-one computer-aided standardized questionnaire to collect demographic variables and information regarding potential predictors of ESCC. Candidate predictors, which were selected based on literature review, included age, gender, socioeconomic status (education level and marital status), cigarette smoking, alcohol consumption, consumption of hot tea, source of drinking water, family history of ESCC, body mass index (BMI), type of fuel used for cooking, exposure to fumes in the kitchen, pesticide exposure, intake of fruit and vegetables, unhealthy dietary habits, and upper GI symptoms in the last 1 month (including dysphagia, retrosternal pain, reflux or heartburn, loss of appetite or dyspepsia, nausea or vomiting, and epigastric pain) (Table S1).

The outcome was defined as the detection of severe dysplasia and above (SDA) of the esophagus, which included severe squamous dysplasia, carcinoma in situ, and ESCC. For the development set and validation set A, it was ascertained based on pathological diagnoses of the biopsy specimens, that were taken from all focal lesions during upper GI endoscopic examination and reviewed independently by two experienced pathologists. The outcome for validation set B was obtained through annual follow-up via active door-to-door interviews and passive linkage with local health insurance claims data, which have been proved to have a sensitivity of over 95% in identifying cancer cases and may be an ideal data source for cancer follow-up [12,13,14].

### 2.3. Statistical Analysis

The chi-squared test and the Kruskal–Wallis rank-sum test were used to compare characteristics of the participants across the three datasets for categorical and continuous variables, respectively.

We used a two-step approach to develop the prediction model. The correlation of each potential predictor with the presence of SDA was first assessed using univariable logistic regression in the development set. Predictors with *p* < 0.05 or *p* < 0.5 and odds ratio > 1.3 were initially selected for multivariable logistic regression. Backward elimination using the Akaike information criterion (AIC) was adopted to determine the final multivariable model, which is to remove predictors that would increase AIC until reaching the smallest AIC. Patients with missing values or other upper GI cancers were excluded from the analysis.

Receiver operating characteristic (ROC) curves were plotted to visually assess the discrimination of the final model. AUCs were calculated according to the observed and predicted values and compared using the DeLong test among the three datasets [15].

We set different screening coverages in the development set to identify ‘high-risk’ individuals in the development set. The highest predicted probability for achieving the desired screening coverage in the development set was then applied to the two validation sets to assess the application performance of the model. We calculated the sensitivity, the average number of endoscopies needed to detect one SDA case, the detection rate, and the detection rate ratio compared to universal screening in each of the three datasets.

All analyses in this study were conducted using R software (version 4.0.2, Ross Ihaka and Robert Gentleman, Auckland, New Zealand). All tests were two-sided and *p* < 0.05 were considered statistically significant.

### 2.4. Ethics Statement

This study was approved by the Institutional Review Board of the Peking University School of Oncology, China. Written informed consent was obtained from all the participants.

## 3. Results

### 3.1. Baseline Characteristics and Outcome

The three datasets showed statistically significant differences in selected characteristics (Table 1). Compared to the general population in validation set B, outpatients recruited from hospitals (development set and validation set A) were more likely to present with dysphagia and retrosternal pain but less likely to have cigarette smoking habits. Participants from Hua County (development set and validation set B) were more likely to have a family history of ESCC and low BMI than participants from Shenzhen (validation set A).

We identified 154 (1.5%) SDA cases among 10,595 participants in the development set, and 49 (0.5%) SDA cases among 9453 participants in validation set A. In validation set B, there were 18 (0.1%), 39 (0.2%), and 66 (0.4%) SDA cases within 1-, 3-, and 5-year period of follow-up, respectively, among the 17,511 participants.

### 3.2. Model Development

Among the candidate variables, seven predictors were selected to construct the new version of the model (Table 2). We provide a simple and easy-to-use calculator (Excel S1) for use in clinical settings to obtain the risk for malignant esophageal lesions based on our model. The formula of the model was as follows:

Y _risk of malignant esophageal lesions_ = 1/(1 + e^ (− (−14.996 + 0.159 × _age_ + 0.531 × _gender_ + 0.639 × _family history_ + 0.412 × _smoking_ + 0.530 × _BMI_ + 1.547 × _dysphagia_ + 0.570 × _retrosternal pain_))) (1)

**Figure 1:**
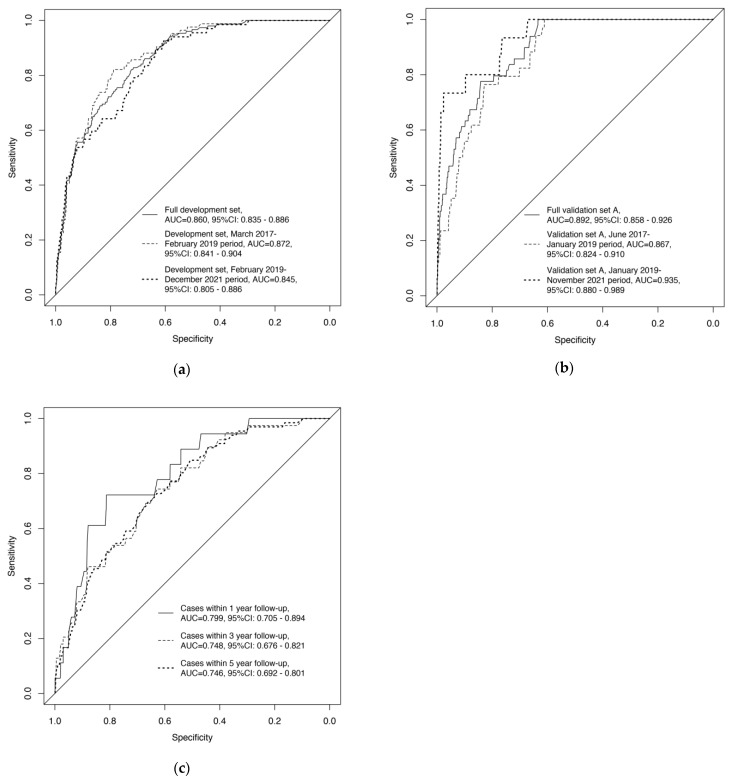
Receiver operating characteristic curves of the improved prediction model for severe dysplasia and above in (**a**) the development set, (**b**) the validation set A, and (**c**) the validation set B. AUC, Area under the curve; CI, confidence interval.

As shown in Figure 1a, the model achieved an AUC of 0.860 (95% confidence interval (CI): 0.835–0.886) in the development set, and AUCs of 0.872 (95% CI: 0.841–0.904) and 0.845 (95% CI: 0.805–0.886) in the subsets of participants recruited in the March 2017–February 2019 period and the February 2019–December 2021 period, respectively.

### 3.3. External Validation of the Model

We observed an overall AUC of 0.892 (95% CI: 0.858–0.926) in validation set A. The model also worked well in the two subsets in this dataset (AUC = 0.867, 95% CI: 0.824–0.910 for the subset recruited in the June 2017–January 2019 period; AUC = 0.935, 95% CI: 0.880–0.989 for the subset recruited in the January 2019–November 2021 period) (Figure 1b).

For validation set B from a general population, the AUCs of the model were 0.799 (95% CI: 0.705–0.894), 0.748 (95% CI: 0.676–0.821), and 0.746 (95% CI: 0.692–0.801) when taking ESCC cases within 1-, 3-, and 5-year follow-up as the outcome, respectively (Figure 1c).

### 3.4. Evaluation of Application Performance of the Model

We set different screening coverages to select ‘high-risk’ individuals in the development set and evaluated the application performance of the model in the two validation sets. In all three datasets, as the cutoff rose and the proportion of individuals defined as at high risk decreased, the detection rate increased notably, reflecting substantial risk enrichment of the model for patients with malignant lesions in the esophagus (Table 3). For example, if only the top 5% of all individuals were referred for endoscopic screening, over 40% of all cases could be detected, and the detection rate would be more than eight times higher than that of universal screening in the development set and validation set A. When the screening coverage was shifted to 30%, over 80% of cases could be detected, with a three-fold increase in the detection rate in the development set and validation set A compared to universal screening. For validation set B, the model could also increase the detection rate by four times (from 0.1% to 0.4%) and two times (from 0.1% to 0.2%) when the top 5% and 30% of the population were screened, respectively.

## 4. Discussion

In this study, we developed an improved version of diagnostic model to predict individualized risk of malignant esophageal lesions in an opportunistic screening scenario. The final model containing seven predictors demonstrated high discrimination ability in the development set as well as in two external validation sets. This model exhibited remarkable generalizability and potential for application in opportunistic screening for ESCC.

For the new model in the present study, seven predictors were selected and most of them were well-recognized risk factors for ESCC. Among these predictors, we included two upper GI symptoms, dysphagia and retrosternal pain. In the traditional epidemiologic concept, screening targets asymptomatic individuals at precancerous stage in a given population. However, disease-related symptoms could not be simply distinguished as positive or none, since it is usually a continuous and gradual process from the completely asymptomatic phase to obvious symptoms that make the patients seek for medical services on their own. Patients who have not consulted healthcare providers for early warning signs, i.e., at preclinical stage, are not necessarily ‘asymptomatic’. In rural China, for example, many ESCC patients delayed seeking medical services until symptoms were quite obvious because of limited socioeconomic status and poor health awareness [16]. In this study, about 5% of the general population (validation set B) had self-reported dysphagia and retrosternal pain. Hence, another fundamental goal of opportunistic screening in this population would be to diagnose patients with symptoms as early as possible to achieve ‘downstaging’ effects [17]. We further conducted a stratification analysis in patients with or without the two ESCC-related symptoms in the development set (Table S2), and significantly increased detection rates were observed in both subgroups, suggesting that this model would provide homogeneous performance in these two subgroups. Additionally, these symptoms may also occur in cases of adenocarcinoma of the esophagogastric junction (AEG). Therefore, we tried to use our model to predict the risk of AEG and the results showed good predictive accuracy (Figure S1). However, due to the differences in etiology, epidemiology, and histology between ESCC and AEG, a model specifically established for AEG would be warranted in the future.

Compared with the original version of the model, two new variables, namely family history of ESCC and gender, were introduced in the present model. Family history has been reported as an important risk factor in most previous epidemiological studies of ESCC, particularly in China. People with a positive family history of ESCC were 1.5 to 2.5 times more likely to have/develop ESCC probably because of shared lifestyle and/or genetic susceptibility [9,18,19,20,21]. Family history was a key predictor in another model we constructed for the identification of high-risk individuals in the general population in high-risk areas of rural China [20]. Gender, the other new predictor, is also a widely recognized risk factor for ESCC. It has been reported that the risk of having malignant esophageal lesions for males was ~1.5 times higher than that for females in high-incidence areas in China, such as the Taihang Mountain area [9,13,22]. The gender difference may be even larger in non-high-risk areas, with the risk of malignant esophageal lesions in males 3 to 4 times higher than that in females [3,13]. After adding these two new predictors, we observed statistically significant improvement of the model in validation set A (Figure S2b), and slightly improved performance, although not statistically significant, in the development set (Figure S2a) and validation set B (Figure S2c) as compared with the original version. This demonstrates the contribution of these new predictors, especially in non-high-risk areas.

We completed multidimensional validation of the model in two independent external populations, which was essential for considering/recommending application of the model in clinical practice [23]. Validation set A was established in a clinical setting, the same as the development set, but in a geographically separated region markedly different from Hua County in population structure. The excellent discrimination ability of this model in this external validation dataset suggested the outstanding generalizability of the model when applied in regions and populations different from the settings where it was developed. Validation set B, which was the control group in a large-scale RCT, recruited participants from the general population in the same region as the development set. Since the outcome in validation set B was ascertained in follow-up, the cases were those which had finally progressed to the cancerous stage, the very target of screening efforts [17]. Although the AUCs when predicting long-term outcomes were slightly lower than that in the two clinical datasets, this model showed generally good discrimination in the general population. The performance of the model in these external populations demonstrated its great potential to be applied in real-world ESCC opportunistic screening programs.

In real-world screening programs for ESCC, the optimal cutoff to define ‘high-risk individuals’ in a certain population should be carefully determined in advance. This decision must be made based on overall consideration of resource availability, population coverage, and the capacity of endoscopic examination in local healthcare facilities. To facilitate decision-making in varied scenarios, we set different cutoffs for risk stratification and evaluated the application performance of the model for each cutoff, rather than simply dividing the population into various risk groups using fixed cutoff points. In situations where the screening program aims to increase the detection rate of malignant lesions to achieve the highest public health benefit as possible with limited resources, we would recommend a higher risk cutoff. For example, the risk cutoff of 0.0585372 may be used to identify the top 5% of the whole population that are predicted to be at the highest risk, for whom endoscopic screening would be recommended. With that cutoff, our model could achieve a detection rate of 12.1% (8.2 times higher than that in universal screening) and reduce the number of endoscopies needed to detect one SDA from 68 to 8. This would greatly relieve the burden on local healthcare system. In contrast, a lower cutoff could be adopted if the human, medical, and financial resources are adequate; for example, a risk cutoff of 0.0185875 to identify 20% of the population for screening. In this case, the screening program could achieve a detection rate of 5.3%, 3.6-fold higher than that in universal screening, and could detect one SDA in every 19 endoscopic examinations on average. In the scenario where the priority of the screening program is to detect all cancer cases, we could still avoid over 30% of screening examinations compared with universal screening by adopting a cutoff of 0.0022627.

As a pre-screening tool prior to the screening examinations, our model can be used, theoretically, in combination with any screening technique for esophageal cancer. Among the screening techniques currently available, upper gastrointestinal endoscopy is the gold standard and has been widely used in China and worldwide. Other screening techniques to detect esophageal cancer such as cytosponge have been reported to be simple and low-cost. The application performance of our model in combination with other screening techniques needs to be further investigated in the future based on real-world data.

A limitation of the present study should also be noted. Although this is a multi-center real-world study with a large sample size, more extensive validations and calibrations in other Chinese and non-Chinese populations are needed to confirm the generalizability and robustness of the model.

## 5. Conclusions

In summary, we developed and externally validated an improved version of the diagnostic model predicting the risk of malignant esophageal lesions for opportunistic screening of ESCC. This easy-to-use questionnaire-based risk stratification tool may be readily integrated into smart portable terminals and social media platforms, thereby greatly promoting the uptake of cancer screening programs through self-risk-assessment and self-health-management among the public. As such, high-risk individuals in the general population may for the first time be empowered to act as the initiator and decision-maker for cancer screening examination, in contrast to the traditional cancer screening strategy in which the leading role has long been taken by the government or healthcare facilities.

## Figures and Tables

**Table 1:** Selected characteristics of individuals in the development set, validation set A, and validation set B.

Variables ^a,b^	Development Set, *n* (%)	Validation Set A, *n* (%)	Validation Set B, *n* (%)	*p* Value ^c^
*n*	10,595	9453	17,511	
Age (years), median (interquartile range)	55 (50, 62)	55 (50, 60)	57 (50, 62)	<0.01
Gender				
Female	6190 (58.4)	5195 (55.0)	9010 (51.5)	<0.01
Male	4405 (41.6)	4258 (45.0)	8501 (48.5)	
Family history of esophageal squamous cell carcinoma				
No	9338 (88.1)	9150 (96.8)	15,904 (90.8)	<0.01
Yes	1257 (11.9)	303 (3.2)	1607 (9.2)	
Cigarette smoking ^d^				
No	7864 (74.2)	6857 (72.5)	11,527 (65.8)	<0.01
Yes	2730 (25.8)	2596 (27.5)	5984 (34.2)	
Body mass index				
>22 kg/m^2^	8430 (81.8)	6050 (64.1)	14,984 (85.9)	<0.01
≤22 kg/m^2^	1877 (18.2)	3392 (35.9)	2465 (14.1)	
Dysphagia ^e^				
No	8642 (81.6)	8197 (86.7)	16,635 (95.0)	<0.01
Yes	1952 (18.4)	1256 (13.3)	876 (5.0)	
Retrosternal pain ^e^				
No	8425 (79.5)	7884 (83.4)	16,770 (95.8)	<0.01
Yes	2169 (20.5)	1569 (16.6)	741 (4.2)	

^a^ Variables were selected by a 2-step selection method in which all candidate predictors were first evaluated in univariable logistic regression models and those with *p* < 0.05 or *p* < 0.5 and odds ratio > 1.3 were subjected to multivariable logistic regression models. The Akaike information criterion was used to determine the final predictor pattern. ^b^ The sum in each variable may not be equal to the total number of participants in each dataset due to missing values. ^c^ *p* values were obtained from the chi-squared test and the Kruskal–Wallis rank-sum test for categorical and continuous variables respectively. ^d^ Cigarette smoking was defined as a smoking history of at least 18 packs of cigarettes per year. ^e^ Positive symptoms were defined as occasional or frequent self-reported symptoms in the previous 1 month.

**Table 2:** Predictors and regression coefficients of the prediction model for severe dysplasia and above (SDA) generated from the development set.

Predictors ^a,b^	Non-SDA/SDA	Univariable OR (95% CI)	Multivariable OR ^c^ (95% CI)	Multivariable Coefficients (95% CI)
Age (years)	-	1.18 (1.14 to 1.21)	1.17 (1.14 to 1.21)	0.159 (0.130 to 0.189)
Gender				
Female	5941/61	Reference	Reference	Reference
Male	4110/90	2.13 (1.54 to 2.96)	1.70 (1.07 to 2.71)	0.531 (0.066 to 0.996)
Family history of esophageal squamous cell carcinoma				
No	8876/120	Reference	Reference	Reference
Yes	1175/31	1.95 (1.31 to 2.91)	1.89 (1.24 to 2.88)	0.639 (0.219 to 1.058)
Cigarette smoking ^d^				
No	7524/86	Reference	Reference	Reference
Yes	2527/65	2.25 (1.63 to 3.12)	1.51 (0.95 to 2.41)	0.412 (−0.055 to 0.879)
Body mass index				
>22 kg/m^2^	8245/104	Reference	Reference	Reference
≤22 kg/m^2^	1806/47	2.06 (1.46 to 2.92)	1.70 (1.18 to 2.44)	0.530 (0.167 to 0.892)
Dysphagia ^e^				
No	8245/72	Reference	Reference	Reference
Yes	1806/79	5.01 (3.63 to 6.92)	4.70 (3.32 to 6.65)	1.547 (1.200 to 1.894)
Retrosternal pain ^e^				
No	8020/92	Reference	Reference	Reference
Yes	2031/59	2.53 (1.82 to 3.52)	1.77 (1.24 to 2.52)	0.570 (0.214 to 0.926)
Intercept	-	-	-	−14.996 (−16.868 to −13.125)

Abbreviation: OR, odds ratio; CI, confidence interval. ^a^ Predictors were selected by a 2-step selection method in which all candidate predictors were first evaluated in univariable logistic regression models and those with *p* < 0.05 or *p* < 0.5 and odds ratio > 1.3 were subjected to multivariable logistic regression models. The Akaike information criterion was used to determine the final predictor pattern. ^b^ Participants with missing values were excluded from the multivariable analysis. ^c^ Odds ratio = exp (coefficient). ^d^ Cigarette smoking was defined as a smoking history of at least 18 packs of cigarettes per year. ^e^ Positive symptoms were defined as occasional or frequent self-reported symptoms in the previous 1 month.

**Table 3:** Evaluation of application performance of the prediction model for different screening coverages to detect severe dysplasia and above (SDA) cases.

Cutoffs ^a^	Development Set ^b^ (*n* = 10,202)			Validation Set A ^b^ (*n* = 9340)			Validation Set B ^c^ (*n* = 17,442)		
	Screening Coverage, % (Sensitivity of Screening, %)	Average No. of Endoscopies to Detect one SDA Case	Detection Rate in the Screening, % (Detection Rate Ratio Compared to Universal Screening)	Screening Coverage, % (Sensitivity of Screening, %)	Average No. of Endoscopies to Detect one SDA Case	Detection Rate in the Screening, % (Detection Rate Ratio Compared to Universal Screening)	Screening Coverage, % (Sensitivity of Screening, %)	Average No. of Endoscopies to Detect one SDA Case	Detection Rate in the Screening, % (Detection Rate Ratio Compared to Universal Screening)
0.1573189	1.0 (13.2)	5	19.0 (12.9)	0.7 (20.4)	7	14.9 (28.5)	0.2 (5.6)	30	3.3 (32.4)
0.1103435	2.0 (19.2)	7	14.1 (9.5)	1.4 (30.6)	9	11.6 (22.2)	0.4 (5.6)	67	1.5 (14.5)
0.0846979	3.0 (25.2)	8	12.4 (8.4)	2.1 (34.7)	12	8.5 (16.1)	0.6 (5.6)	112	0.9 (8.7)
0.0690726	4.0 (33.1)	8	12.2 (8.3)	2.9 (36.7)	15	6.6 (12.6)	1.0 (5.6)	183	0.5 (5.3)
0.0585372	5.0 (41.7)	8	12.1 (8.2)	3.6 (38.8)	18	5.7 (10.8)	1.6 (5.6)	273	0.4 (3.6)
0.0520559	6.0 (43.7)	9	10.7 (7.2)	4.2 (44.9)	18	5.6 (10.8)	2.1 (11.1)	180	0.6 (5.4)
0.0469379	7.0 (49.0)	10	10.3 (6.9)	4.9 (46.9)	20	5.0 (9.6)	2.5 (11.1)	219	0.5 (4.4)
0.0424342	8.0 (53.6)	10	9.9 (6.7)	5.8 (46.9)	23	4.3 (8.1)	3.2 (16.7)	186	0.5 (5.2)
0.0383463	9.0 (55.6)	11	8.9 (6.0)	6.7 (53.1)	24	4.2 (8.0)	4.2 (16.7)	246	0.4 (4.0)
0.0347929	10.0 (55.6)	12	8.2 (5.5)	7.2 (57.1)	24	4.2 (8.0)	4.6 (16.7)	269	0.4 (3.6)
0.0185875	20.0 (72.2)	19	5.3 (3.6)	16.1 (77.6)	40	2.5 (4.8)	15.0 (61.1)	238	0.4 (4.1)
0.0116659	30.0 (82.8)	24	4.1 (2.8)	24.9 (79.6)	60	1.7 (3.2)	26.2 (72.2)	353	0.3 (2.8)
0.0076619	40.0 (91.4)	30	3.4 (2.3)	34.0 (93.9)	69	1.4 (2.8)	37.4 (77.8)	468	0.2 (2.1)
0.0050037	50.0 (96.0)	35	2.8 (1.9)	45.2 (100.0)	86	1.2 (2.2)	49.2 (88.9)	539	0.2 (1.8)
0.0032098	60.0 (98.7)	41	2.4 (1.6)	56.9 (100.0)	109	0.9 (1.8)	58.0 (94.4)	597	0.2 (1.6)
0.0022627	70.0 (100.0)	47	2.1 (1.4)	67.8 (100.0)	129	0.8 (1.5)	68.1 (94.4)	701	0.1 (1.4)
0.0014971	80.0 (100.0)	54	1.8 (1.2)	79.3 (100.0)	151	0.7 (1.3)	76.2 (100.0)	741	0.1 (1.3)
0.0009304	90.0 (100.0)	61	1.6 (1.1)	90.3 (100.0)	172	0.6 (1.1)	87.7 (100.0)	853	0.1 (1.1)
0.0003394	100.0 (100.0)	68	1.5 (1.0)	99.9 (100.0)	191	0.5 (1.0)	99.6 (100.0)	969	0.1 (1.0)

^a^ Cutoffs were selected as the highest predicted probabilities that ensured corresponding screening coverage in the development set. ^b^ Participants with missing values were excluded. ^c^ Cases were those that developed esophageal squamous cell carcinoma within 1-year follow-up. Participants with missing values were excluded.

## Data Availability

Data used in this study are available from the corresponding authors upon reasonable request.

## Supplementary Materials

The following supporting information can be downloaded at: https://www.mdpi.com/article/10.3390/cancers14235945/s1, Excel S1: Calculator for the risk of malignant esophageal lesions; Figure S1: Receiver operating characteristic curves of the improved version of the diagnostic model for adenocarcinoma of the esophagogastric junction in (**a**) the development set, (**b**) validation set A, and (**c**) validation set B; Figure S2: Receiver operating characteristic curves of the improved version and original version of the diagnostic model for severe dysplasia and above in (**a**) the development set, (**b**) validation set A, and (**c**) validation set B; Table S1: Distribution of all the candidate predictors in the development set, validation set A, and validation set B; Table S2: Application performance of the diagnostic model for different screening coverages to detect severe dysplasia and above (SDA) cases in the development set stratified by symptom.

## Abbreviations

EC: esophageal cancer; ESCC, esophageal squamous cell carcinoma; AUC, area under the receiver operating characteristic curve; RCT, randomized controlled trial; GI, gastrointestinal; ESECC, the Efficacy of endoscopic Screening for Esophageal Cancer in China trial; BMI, body mass index; SDA, severe dysplasia and above; AIC, Akaike information criterion; ROC, receiver operating characteristic; OR, odds ratio; CI, confidence interval; AEG, adenocarcinoma of the esophagogastric junction.

## References

[1] Sung H., Ferlay J., Siegel R.L., Laversanne M., Soerjomataram I., Jemal A., Bray F. (2021). Global cancer statistics 2020: GLOBOCAN estimates of incidence and mortality worldwide for 36 cancers in 185 countries. CA Cancer J. Clin..

[2] Bray F., Ferlay J., Soerjomataram I., Siegel R., Torre L., Jemal A. (2018). Global cancer statistics 2018: GLOBOCAN estimates of incidence and mortality worldwide for 36 cancers in 185 countries. CA Cancer J. Clin..

[3] Rongshou Z., Siwei Z., Hongmei Z., Shaoming W., Kexin S., Ru C., Li L., Wenqiang W., Jie H. (2022). Cancer incidence and mortality in China, 2016. J. Natl. Cancer Cent..

[4] Zeng H., Zheng R., Guo Y., Zhang S., Zou X., Wang N., Zhang L., Tang J., Chen J., Wei K. (2015). Cancer survival in China, 2003-2005: A population-based study. Int. J. Cancer.

[5] He Z., Ke Y. (2020). Precision screening for esophageal squamous cell carcinoma in China. Chin. J. Cancer Res..

[6] Wei W., Chen Z., He Y., Feng H., Hou J., Lin D., Li X., Guo C., Li S., Wang G. (2015). Long-Term Follow-Up of a Community Assignment, One-Time Endoscopic Screening Study of Esophageal Cancer in China. J. Clin. Oncol..

[7] Liu M., He Z., Guo C., Xu R., Li F., Ning T., Pan Y., Li Y., Ding H., Zheng L. (2019). Effectiveness of Intensive Endoscopic Screening for Esophageal Cancer in China: A Community-Based Study. Am. J. Epidemiol..

[8] Chen R., Liu Y., Song G., Li B., Zhao D., Hua Z., Wang X., Li J., Hao C., Zhang L. (2021). Effectiveness of one-time endoscopic screening programme in prevention of upper gastrointestinal cancer in China: A multicentre population-based cohort study. Gut.

[9] He Z., Liu Z., Liu M., Guo C., Xu R., Li F., Liu A., Yang H., Shen L., Wu Q. (2019). Efficacy of endoscopic screening for esophageal cancer in China (ESECC): Design and preliminary results of a population-based randomised controlled trial. Gut.

[10] Chen W., Zeng H., Chen R., Xia R., Yang Z., Xia C., Zheng R., Wei W., Zhuang G., Yu X. (2017). Evaluating efficacy of screening for upper gastrointestinal cancer in China: A study protocol for a randomized controlled trial. Chin. J. Cancer Res..

[11] Liu Z., Guo C., He Y., Chen Y., Ji P., Fang Z., Li F., Tang Y., Chen X., Xiao P. (2020). A clinical model predicting the risk of esophageal high-grade lesions in opportunistic screening: A multicenter real-world study in China. Gastrointest Endosc..

[12] Tian H., Xu R., Li F., Guo C., Zhang L., Liu Z., Liu M., Pan Y., He Z., Ke Y. (2019). Identification of cancer patients using claims data from health insurance systems: A real-world comparative study. Chin. J. Cancer Res..

[13] Tian H., Yang W., Hu Y., Liu Z., Chen L., Lei L., Zhang F., Cai F., Xu H., Liu M. (2020). Estimating cancer incidence based on claims data from medical insurance systems in two areas lacking cancer registries in China. EClinicalMedicine.

[14] Shi C., Liu M., Liu Z., Guo C., Li F., Xu R., Liu F., Liu Y., Li J., Cai H. (2019). Using health insurance reimbursement data to identify incident cancer cases. J. Clin. Epidemiol..

[15] DeLong E., DeLong D., Clarke-Pearson D. (1988). Comparing the areas under two or more correlated receiver operating characteristic curves: A nonparametric approach. Biometrics.

[16] Wang J., Liu F., Gao H., Wei W., Zhang X., Liang Y., Cheng Y. (2008). The symptom-to-treatment delay and stage at the time of treatment in cancer of esophagus. Jpn. J. Clin. Oncol..

[17] He Z., Ke Y. (2020). Response. Gastrointest Endosc..

[18] Chen T., Cheng H., Chen X., Yuan Z., Yang X., Zhuang M., Lu M., Jin L., Ye W. (2015). Family history of esophageal cancer increases the risk of esophageal squamous cell carcinoma. Sci. Rep..

[19] Liu M., Liu Z., Liu F., Guo C., Xu R., Li F., Liu A., Yang H., Zhang S., Shen L. (2020). Absence of Iodine Staining Associates with Progression of Esophageal Lesions in a Prospective Endoscopic Surveillance Study in China. Clin. Gastroenterol. Hepatol..

[20] Liu M., Liu Z., Cai H., Guo C., Li X., Zhang C., Wang H., Hang D., Liu F., Deng Q. (2017). A Model to Identify Individuals at High Risk for Esophageal Squamous Cell Carcinoma and Precancerous Lesions in Regions of High Prevalence in China. Clin. Gastroenterol. Hepatol..

[21] Su Z., Zou G., Mao Y., OuYang P., Cao X., Xie F., Li Q. (2019). Prognostic impact of family history of cancer in Southern Chinese patients with esophageal squamous cell cancer. J. Cancer.

[22] Tian H., Hu Y., Li Q., Lei L., Liu Z., Liu M., Guo C., Liu F., Liu Y., Pan Y. (2021). Estimating cancer survival and prevalence with the Medical-Insurance-System-based Cancer Surveillance System (MIS-CASS): An empirical study in China. EClinicalMedicine.

[23] Toll D., Janssen K., Vergouwe Y., Moons K. (2008). Validation, updating and impact of clinical prediction rules: A review. J. Clin. Epidemiol..

