# Supplementary material for: Development and External Validation of an Improved Version of the Diagnostic Model for Opportunistic Screening of Malignant Esophageal Lesions
Source: Cancers (Basel). 2022 Nov 30;14(23):5945. doi: 10.3390/cancers14235945 (PMC9737355; doi:10.3390/cancers14235945)
Supplement: Supplementary file 1 [file cancers-14-05945-s001.zip › Supplementary materials.pdf]

Supplementary materials list:
Excel S1; Figure S1; Figure S2; Table S1; Table S2.

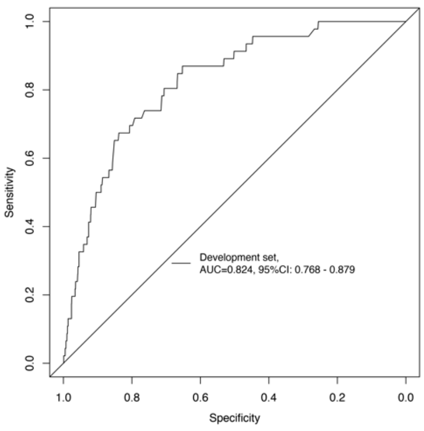

(**a**)

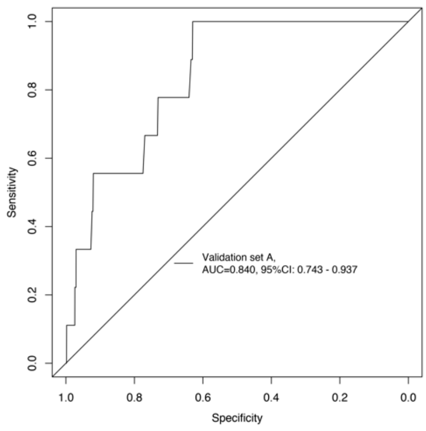

(**b**)

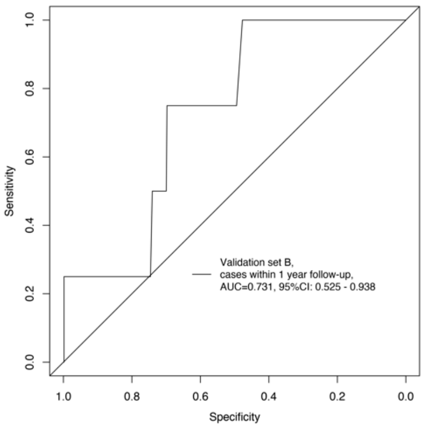

(**c**)

Figure S1. Receiver operating characteristic curves of the improved version of the diagnostic model for adenocarcinoma of the esophagogastric junction in (**a**) the development set, (**b**) the validation set A, and (**c**) the validation set B. AUC, Area under the curve; CI, confidence interval.

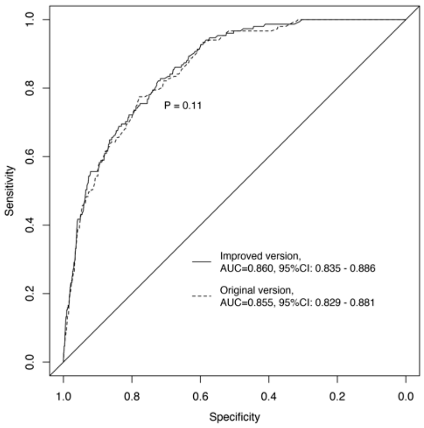

(**a**)

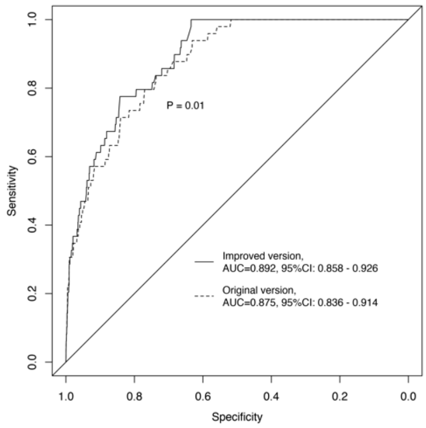

(**b**)

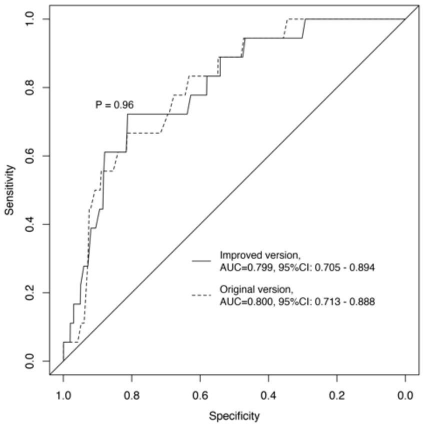

(**c**)

Figure S2. Receiver operating characteristic curves of the improved version and original version of the diagnostic model for severe dysplasia and above in (**a**) the development set, (**b**) the validation set A, and (**c**) the validation set B. AUC, Area under the curve; CI, confidence interval.

Table S1. Distribution of all the candidate predictors in the development set, validation set A, and validation set B.

Variables [a]	Development set [b], n (%)	Validation set A [b], n (%)	Validation set B, n (%)	*p* value [c]
N	10595	9453	17511	
Age (years), median (inter quartile range)	55 (50, 62)	55 (50, 60)	57 (50, 62)	<0.01
Gender				
Female	6190 (58.4)	5195 (55.0)	9010 (51.5)	<0.01
Male	4405 (41.6)	4258 (45.0)	8501 (48.5)	
Education				
Middle school or above	5114 (48.3)	6801 (71.9)	7987 (45.6)	<0.01
Primary school or below	5480 (51.7)	2652 (28.1)	9524 (54.4)	
Marital status				
Married	685 (6.5)	222 (2.3)	1011 (5.8)	<0.01
Unmarried	9909 (93.5)	9231 (97.7)	16500 (94.2)	
No. of family members				
1	284 (2.7)	339 (3.6)	461 (2.6)	
2	2555 (24.1)	1907 (20.2)	5118 (29.2)	<0.01
≥ 3	7753 (73.2)	7196 (76.2)	11931 (68.1)	
Family history of esophageal squamous cell carcinoma				
No	9338 (88.1)	9150 (96.8)	15904 (90.8)	<0.01
Yes	1257 (11.9)	303 (3.2)	1607 (9.2)	
Cigarette smoking [d]				
No	7864 (74.2)	6857 (72.5)	11527 (65.8)	<0.01
Yes	2730 (25.8)	2596 (27.5)	5984 (34.2)	
Alcohol consumption [e]				
No	8692 (82.0)	7932 (83.9)	13294 (75.9)	<0.01
Yes	1903 (18.0)	1521 (16.1)	4217 (24.1)	
Body mass index				
> 22 kg/m^2	8430 (81.8)	6050 (64.1)	14984 (85.9)	<0.01
≤ 22 kg/m^2	1877 (18.2)	3392 (35.9)	2465 (14.1)	
Eating irregularity				

No	9270 (87.5)	7736 (81.8)	16005 (91.4)	<0.01
Yes	1325 (12.5)	1717 (18.2)	1506 (8.6)	
Eating speed				
Slow	1713 (16.2)	1302 (13.8)	3190 (18.2)	<0.01
Fast	8882 (83.8)	8151 (86.2)	14321 (81.8)	
Preference of food temperature				
Cool	1205 (11.4)	1319 (14.0)	2341 (13.4)	<0.01
Hot	9390 (88.6)	8134 (86.0)	15170 (86.6)	
Preference of food hardness				
Soft	3603 (34.0)	3550 (37.6)	6216 (35.5)	<0.01
Hard	6992 (66.0)	5903 (62.4)	11295 (64.5)	
Preference of salty food				
No	2530 (28.4)	3478 (39.3)	4567 (26.1)	<0.01
Yes	6377 (71.6)	5366 (60.7)	12944 (73.9)	
Frequency of vegetable consumption				
Seldom	64 (0.6)	9 (0.1)	319 (1.8)	<0.01
Frequent	10531 (99.4)	9444 (99.9)	17192 (98.2)	
Frequency of fruit consumption				
Seldom	2624 (24.8)	677 (7.2)	3417 (19.5)	<0.01
Frequent	7971 (75.2)	8776 (92.8)	14094 (80.5)	
Frequency of meat consumption				
Seldom	1287 (12.1)	168 (1.8)	4089 (23.4)	<0.01
Frequent	9308 (87.9)	9285 (98.2)	13422 (76.6)	
Fume exposure in the kitchen				
No	5431 (51.3)	6900 (73.0)	7025 (40.1)	<0.01
Yes	5162 (48.7)	2553 (27.0)	10486 (59.9)	
Consumption of leftover food				
No	5396 (50.9)	3314 (35.1)	10248 (58.5)	<0.01
Yes	5199 (49.1)	6139 (64.9)	7263 (41.5)	
Consumption of pickled food				
No	6899 (65.1)	4645 (49.1)	11556 (66.0)	<0.01
Yes	3696 (34.9)	4808 (50.9)	5955 (34.0)	
Dysphagia [f]				
No	8642 (81.6)	8197 (86.7)	16635 (95.0)	<0.01
Yes	1952 (18.4)	1256 (13.3)	876 (5.0)	
Retrosternal pain [f]				
No	8425 (79.5)	7884 (83.4)	16770 (95.8)	<0.01
Yes	2169 (20.5)	1569 (16.6)	741 (4.2)	
Reflux [f]				
No	5446 (51.4)	5046 (53.4)	12425 (71.0)	<0.01
Yes	5148 (48.6)	4407 (46.6)	5086 (29.0)	
Dyspepsia [f]				
No	6277 (59.3)	4836 (51.2)	14012 (80.0)	<0.01
Yes	4317 (40.7)	4617 (48.8)	3499 (20.0)	
Nausea [f]				
No	6624 (62.5)	5631 (59.6)	15835 (90.4)	<0.01
Yes	3970 (37.5)	3822 (40.4)	1676 (9.6)	
Epigastric pain [f]				
No	6285 (59.3)	3693 (39.1)	16013 (91.4)	<0.01
Yes	4309 (40.7)	5760 (60.9)	1498 (8.6)	

[a] Variables were candidate predictors for model derivation.

[b] The sum in each variable may not be equal to the total number of participants in each dataset due to missing values.
[c] *p* values were obtained from the chi-squared test and the Kruskal-Wallis rank-sum test for categorical and continuous variables respectively.
[d] Cigarette smoking was defined as a smoking history of at least 18 packs of cigarettes per year.
[e] Alcohol drinking was defined as drinking Chinese liquor (containing > 40% alcohol) at least twice a week for ≥12 months (consumption of other kinds of alcoholic beverages such as beer and red wine is very rare in the study area).
[f] Positive symptoms were defined as occasional or frequent self-reported symptoms in the previous 1 month.

Table S2. Application performance of the diagnostic model for different screening coverages to detect severe dysplasia and above (SDA) cases in the development set stratified by symptom.

	**Development set (n = 10202)**			**Participants with ≥ 1 symptoms (n = 3271)**			**Participants without symptoms (n = 6931)**		
**Cutoffs [a]**	**Screening coverage, % (sensitivity of screening, %)**	**Average No. of endoscopies to detect one SDA case**	**Detection rate in the screening, % (detection rate ratio compared to universal screening)**	**Screening coverage, % (sensitivity of screening, %)**	**Average No. of endoscopies to detect one SDA case**	**Detection rate in the screening, % (detection rate ratio compared to universal screening)**	**Screening coverage, % (sensitivity of screening, %)**	**Average No. of endoscopies to detect one SDA case**	**Detection rate in the screening, % (detection rate ratio compared to universal screening)**
0.0585372	5.0 (41.7)	8	12.14 (8.2)	14.4 (56.7)	8	12.50 (3.9)	0.7 (8.5)	12	8.51 (12.6)
0.0347929	10.0 (55.6)	12	8.21 (5.5)	24.0 (71.2)	11	9.44 (3.0)	3.4 (21.3)	24	4.18 (6.2)
0.0244539	15.0 (65.6)	15	6.46 (4.4)	30.7 (80.8)	12	8.36 (2.6)	7.6 (31.9)	35	2.84 (4.2)
0.0185875	20.0 (72.2)	19	5.34 (3.6)	38.2 (84.6)	14	7.05 (2.2)	11.5 (44.7)	38	2.64 (3.9)
0.0116659	30.0 (82.8)	24	4.08 (2.8)	49.0 (92.3)	17	5.99 (1.9)	21.0 (61.7)	50	1.99 (2.9)
0.0076619	40.0 (91.4)	30	3.38 (2.3)	61.5 (98.1)	20	5.07 (1.6)	29.9 (76.6)	58	1.74 (2.6)
0.0050037	50.0 (96.0)	35	2.83 (1.9)	72.2 (99.0)	23	4.36 (1.4)	39.9 (89.4)	66	1.52 (2.2)
0.0014971	80.0 (100.0)	54	1.85 (1.2)	95.7 (100.0)	30	3.32 (1.0)	72.9 (100.0)	107	0.93 (1.4)
0.0003394	100.0 (100.0)	68	1.48 (1.0)	100.0 (100.0)	31	3.18 (1.0)	100.0 (100.0)	147	0.68 (1.0)

[a] Cutoffs were selected as the highest predicted probabilities that ensured corresponding screening coverage in the development set.
