# Supplementary figures and images for: Development and External Validation of an Improved Version of the Diagnostic Model for Opportunistic Screening of Malignant Esophageal Lesions
Source: Cancers (Basel). 2022 Nov 30;14(23):5945. doi: 10.3390/cancers14235945 (PMC9737355; doi:10.3390/cancers14235945)

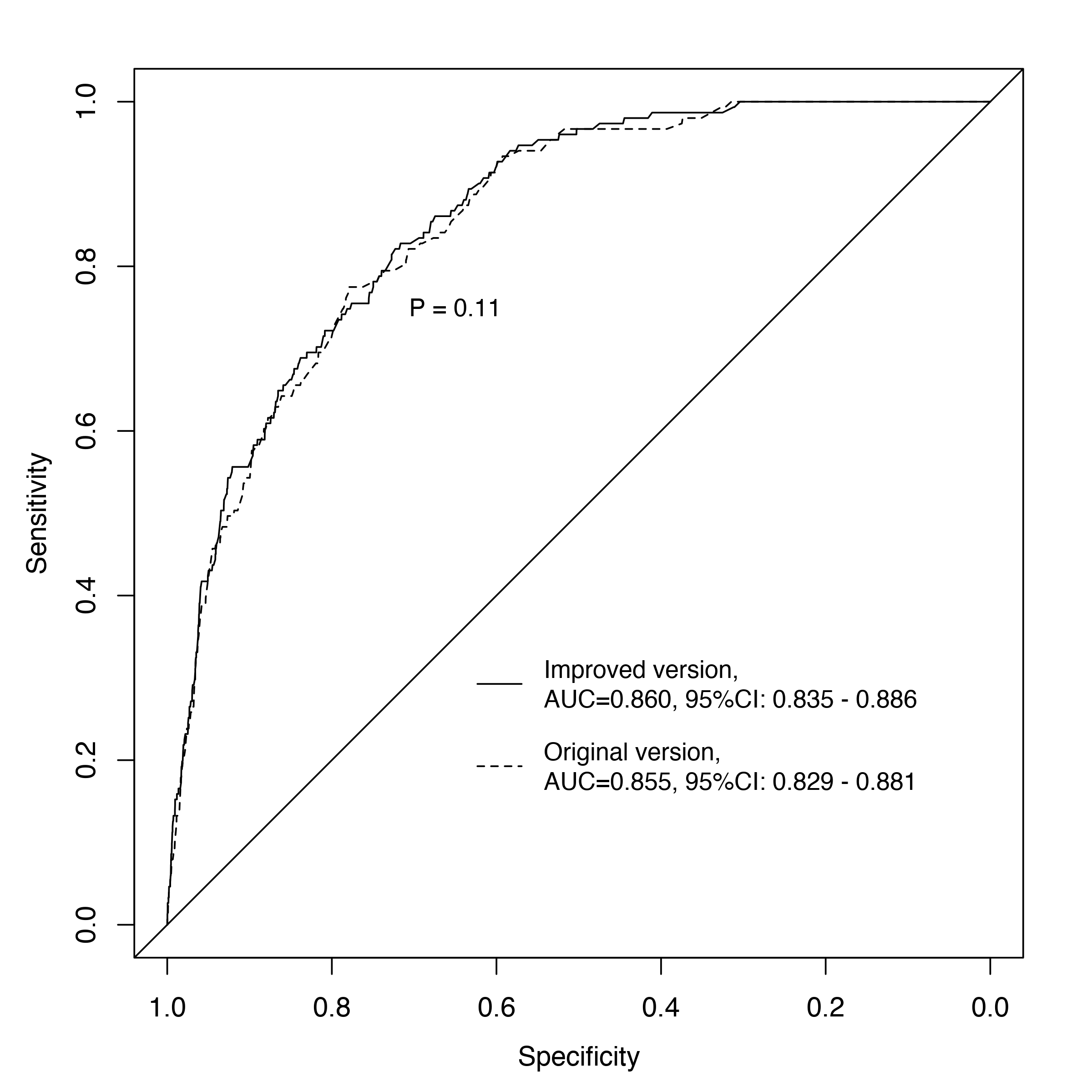

Supplement: Supplementary file 1 [file cancers-14-05945-s001.zip › Figure S1 a.tiff]

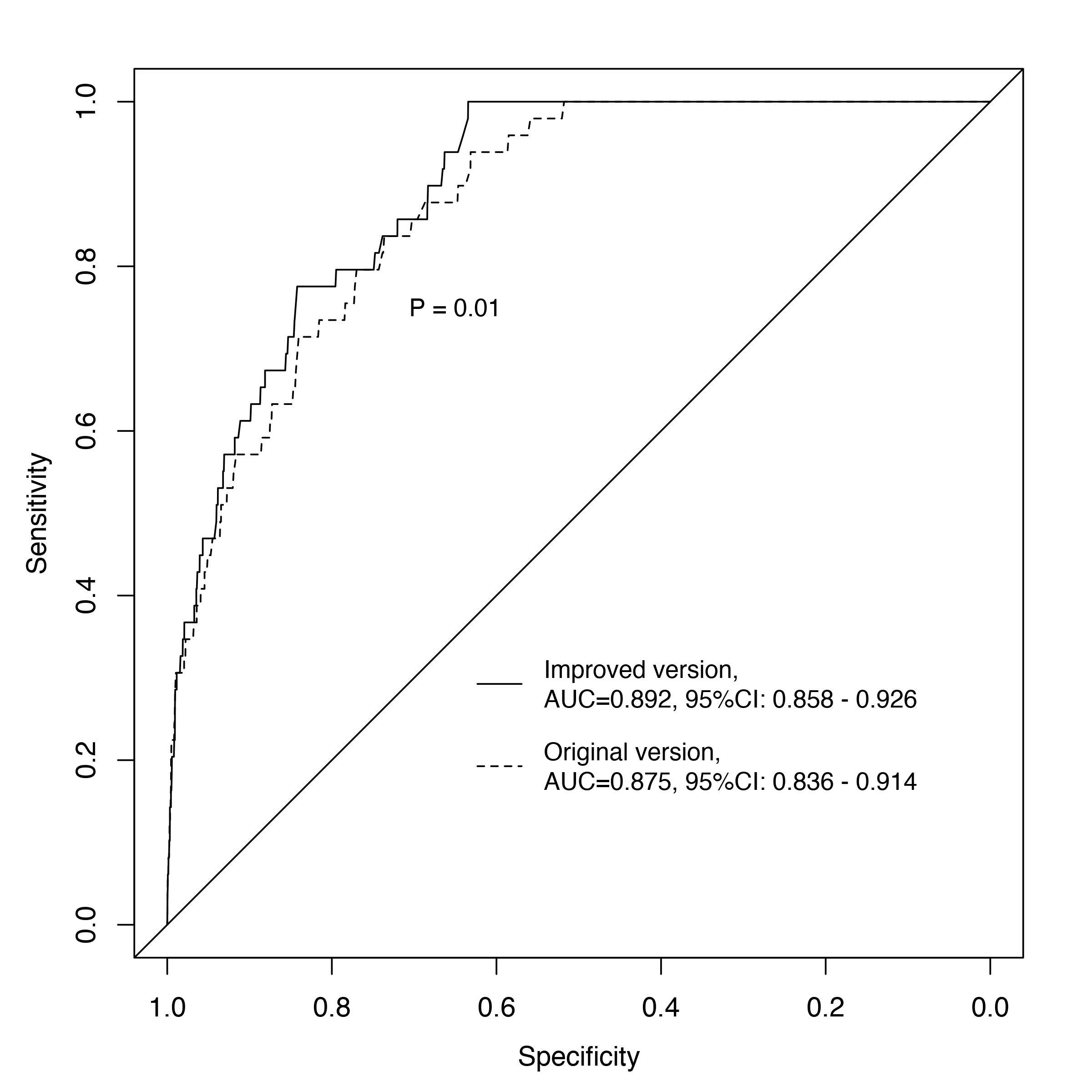

Supplement: Supplementary file 1 [file cancers-14-05945-s001.zip › Figure S1 b.tiff]

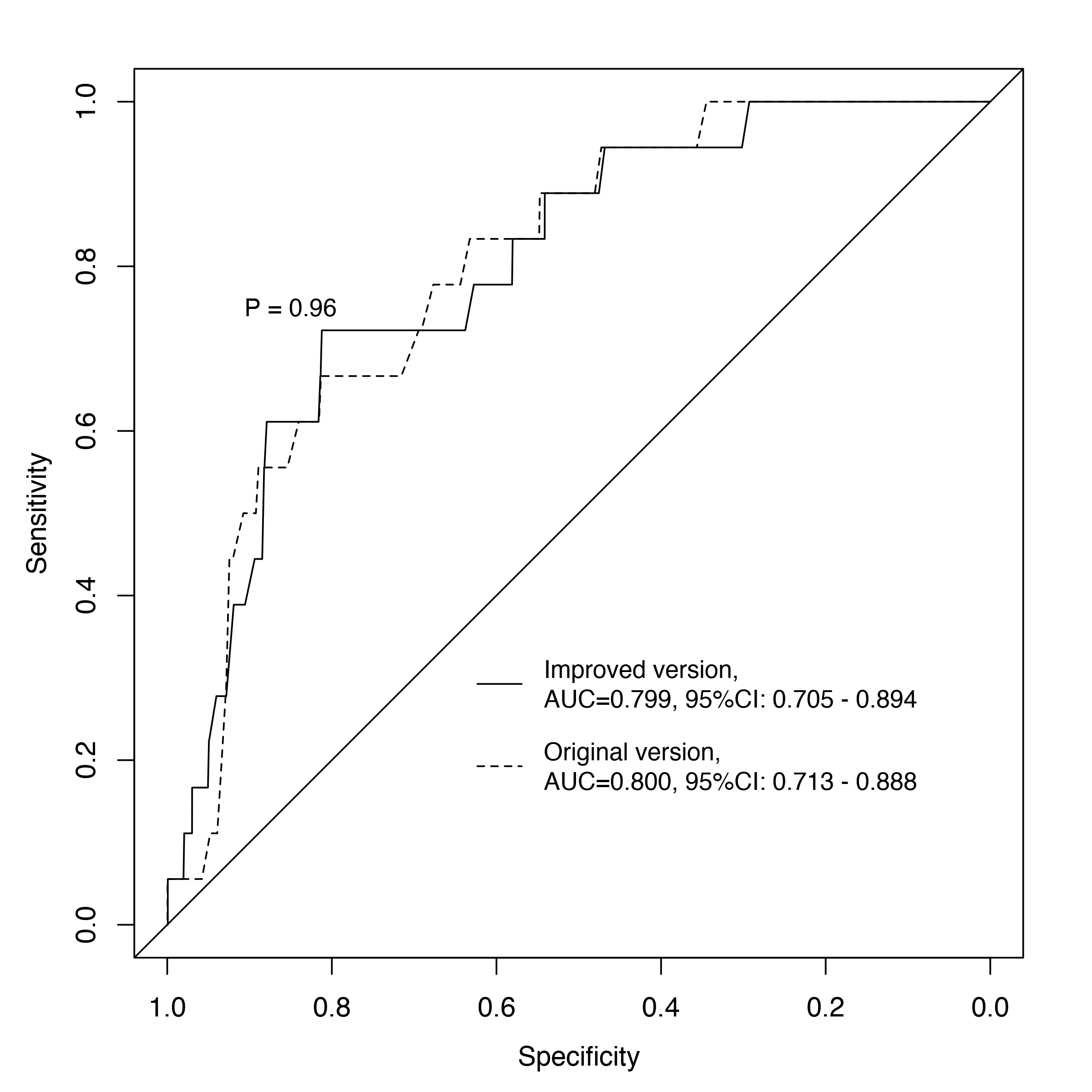

Supplement: Supplementary file 1 [file cancers-14-05945-s001.zip › Figure S1 c.tiff]

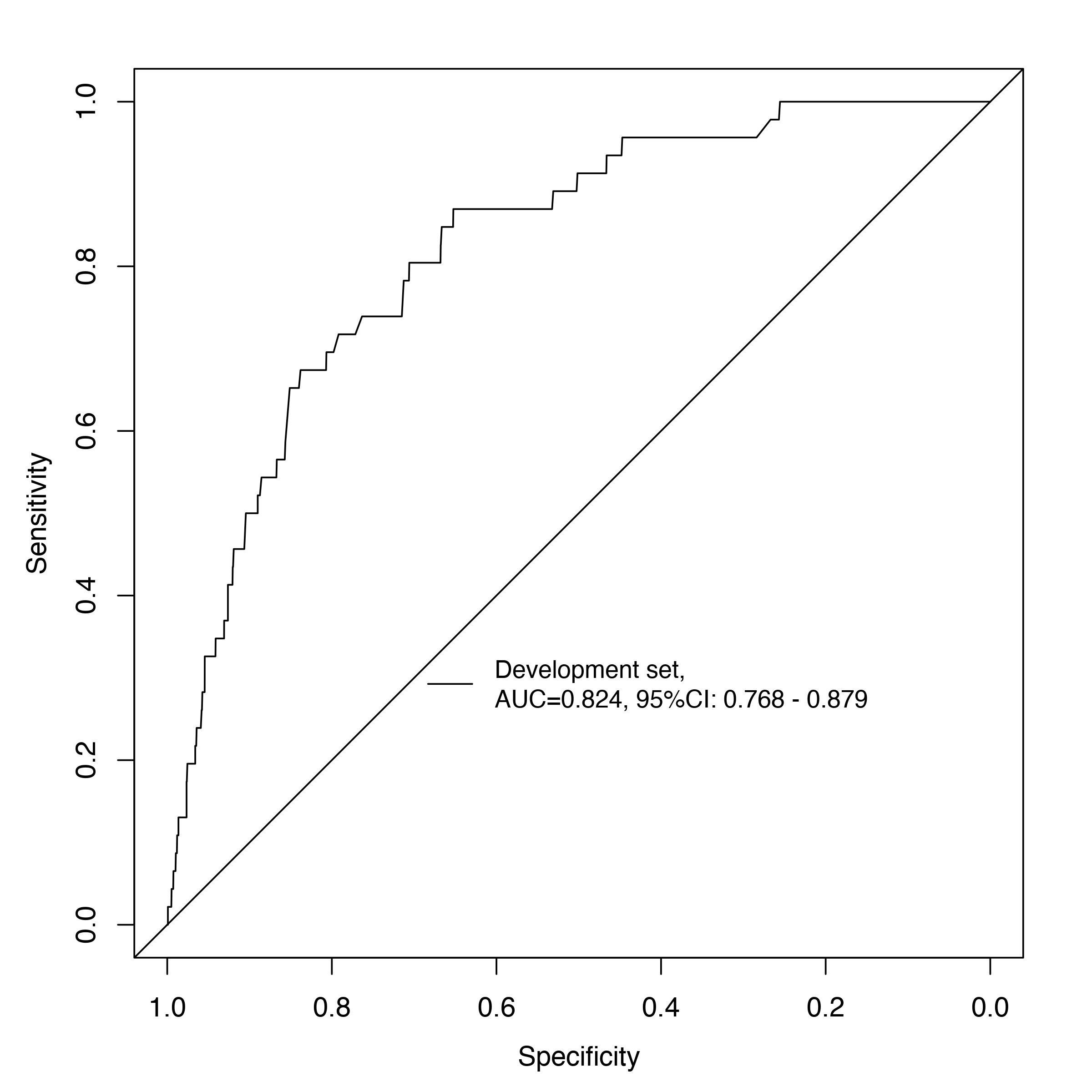

Supplement: Supplementary file 1 [file cancers-14-05945-s001.zip › Figure S2 a.tiff]

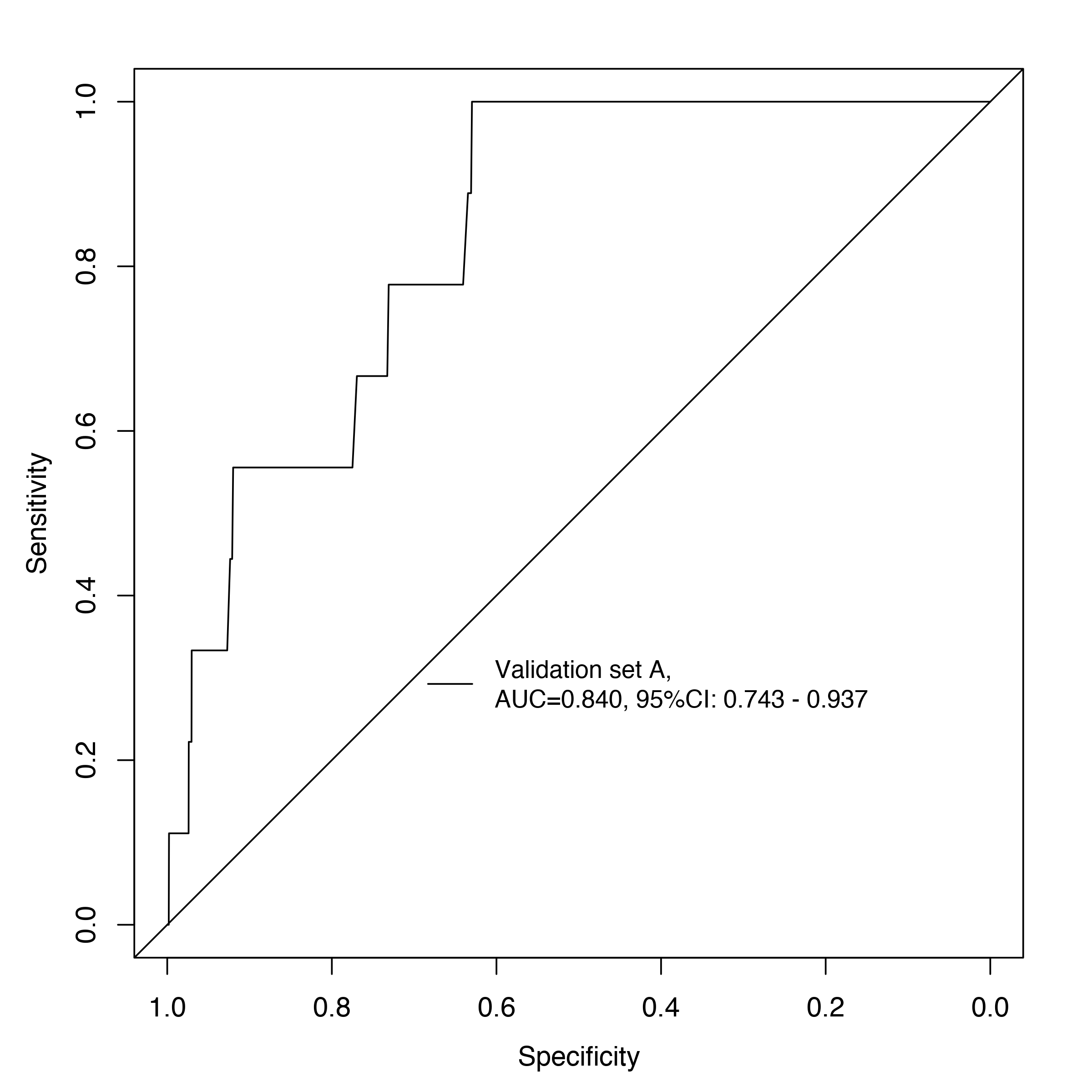

Supplement: Supplementary file 1 [file cancers-14-05945-s001.zip › Figure S2 b.tiff]

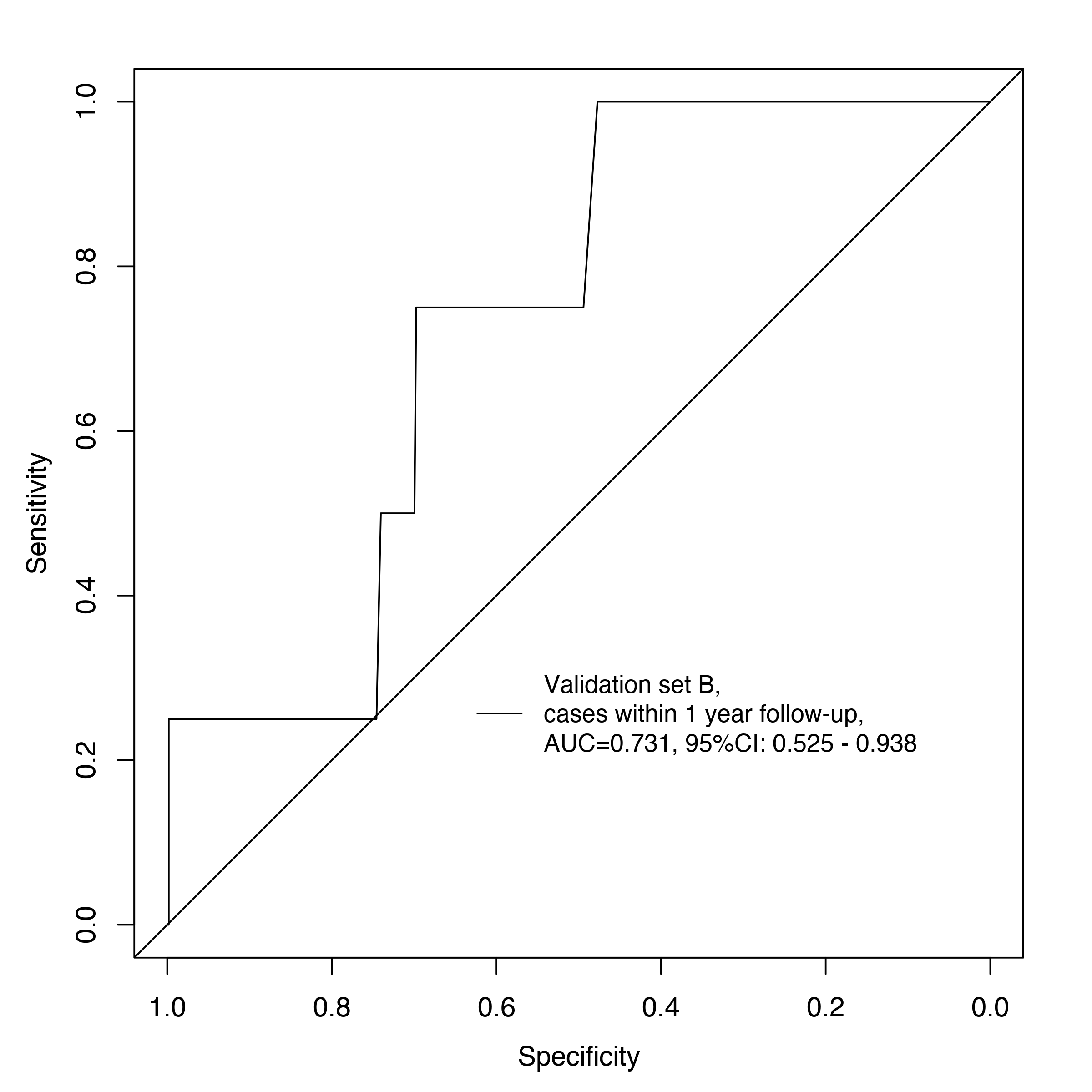

Supplement: Supplementary file 1 [file cancers-14-05945-s001.zip › Figure S2 c.tiff]
